# American Medical Association

**Published:** 1882-09-20

**Authors:** Richard J. Dunglison

**Affiliations:** Treasurer, Secretary Committee of Publication; Philadelphia, Penn.


### *AMERICAN MEDICAL ASSOCIATION.*

PHILADELPHIA, PENN., July, 1882.

DEAR SIR—You are respectfully informed that the Committee of Publication have adopted the following rules, to ensure promptness in the appearance of the forthcoming volume of Transactions (Vol. 33):

1. All addresses and papers read at the recent meeting of the Association, and referred to the Committee of Publication, must be in the hands of the Permanent Secretary before July 31st.

2. The transactions will absolutely go to press August 5th, and all papers or addresses entitled to appear in this volume, not received by July 31st, cannot be inserted.

3. Under no circumstances will the Committee permit new material, different from that in the original manuscript, to be added to the proof-sheets.

4. The Secretary is instructed to send copies of these resolutions to the various medical journals, and to have extra copies struck off, to forward to the contributors of papers to the annual volume.

The following provisions of the by-laws of the Association will be strictly enforced:

"Every paper received by this Association and ordered to be published, and all plates or other means of illustration, shall be considered the exclusive property of the Association, and shall be published and sold for the exclusive benefit of the Association."

"Authors of papers are required to return their proofs within two weeks after their reception; otherwise they will be passed over and omitted from the volume." Yours respectfully,

RICHARD J. DUNGLISON, Treasurer,
Secretary Committee of Publication.

